# EBV protection‐ and susceptibility‐related HLA alleles and EBV status in the Chinese population: A single‐center study

**DOI:** 10.1002/iid3.666

**Published:** 2022-06-21

**Authors:** Dina Suolitiken, Yini Wang, Zhili Jin, Zhao Wang

**Affiliations:** ^1^ Department of Hematology, Beijing Friendship Hospital Capital Medical University Beijing People's Republic of China

**Keywords:** EBV, HLA, infection

## Abstract

**Background:**

Although most adults are infected by Epstein‐Barr virus (EBV), some patients develop highly lethal diseases associated with EBV infection, including EBV‐hemophagocytic lymphohistiocytosis (EBV‐HLH), chronic active EBV infections (CAEBV), and lymphoma, the pathogeneses of which remain to be investigated. The human leukocyte antigen (HLA) complex may be associated with the viral infection pathway, and, therefore, HLA alleles may be associated with EBV‐related diseases and subpopulations of infected cells, studies related to EBV‐associated diseases, and subpopulations of infected cells that were conducted in China are scarce.

**Methods:**

In this study, we analyzed the high‐resolution HLA genotypes of 269 patients with EBV‐associated diseases and 213 EBV‐seronegative hematopoietic stem cell donors using PCR‐SBT assay and elucidated the associations of HLA‐A, ‐B, ‐C, ‐DRB1, and ‐DQB1 alleles with EBV‐associated diseases in the Chinese population, Benjamini–Hochberg correction to adjust for multiple testing. HLA genotypes were also analyzed in patients with EBV‐associated diseases showing EBV‐infected lymphocyte subpopulations.

**Results:**

We found that individuals carrying the following alleles showed the following levels of risks: HLA‐DRB1*11 allele, reduced risk of EBV‐related disease (OR [odds ratio]: 0.56; 95% confidence interval [95% CI]: 0.32–0.99; *p* < .05; Adjust *p* = .71); HLA‐DQB1*06:02 allele, reduced risk (OR: 0.5699; 95% CI: 0.3486–0.9317; *p* < .05; Adjust *p* = .57); and HLA‐B*15:01 allele, increased risk (OR: 1.763; 95% CI: 0.3486–0.9317; *p* < .05; Adjust *p* = .57). Patients with EBV‐associated diseases showing the B*15:01 genotype had a higher risk of T‐cell, NK‐cell, and multicell infections than those with other genotype subgroups.

**Conclusions:**

These findings highlight the importance of HLA genotype for assessing EBV infectivity.

## Graphical abstract

The HLA‐B*15:01 group has a higher rate of EBV infection than other genotype subgroups, B*15:01 is more likely to have non‐B‐cell infections, and the predominant type of EBV‐infected lymphocytes in patients with the B*15:01 genotype should be examined. These findings highlight the importance of HLA genotype for EBV infectivity and that HLA genotype may be used as a marker for some important risk classification

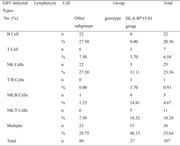

## 1 INTRODUCTION

The major histocompatibility complex (MHC) is a collective term used for a group of genes that encode the major histocompatibility antigens in animals. In humans, the MHC is known as the human leukocyte antigen (HLA) complex, and the main function of the HLA complex is to present antigens from pathogens, tissue‐specific differentiation antigens, or mutated proto‐oncogenes on the cell surface for recognition by T cells in response to infection or malignancy.^1^ HLA acts as a key determinant of resistance to infection. The major HLA Class I genes (HLA‐A, HLA‐B, and HLA‐C) are located on chromosome 6.^2^ The level of polymorphism seen in some genes is very high, with over 3000 variants seen in HLA‐B; this level of variation can be considered hyper‐polymorphic compared to other gene systems.^3^ The evolution of HLA molecular polymorphisms is thought to be the result of selection and adaptation via the modulation of host immunity against infections, tumors, and other diseases.^4^ Thus, there is a strong link between HLA alleles and various diseases, including infections, autoimmune diseases, and tumors.^5^

Latent Epstein‐Barr virus (EBV) infection is present in over 90% of the world's adult population. Social/economic factors and the genomic make‐up of individuals can influence susceptibility to viral infection and the outcome of infection. EBV mainly infects B cells, pharyngeal epithelial cells, NK cells, T cells, and monocytes/macrophages, and it causes different outcomes depending on the immune status of the body, with lifelong postinfection persistence.^6^ Although most people with EBV infection remain asymptomatic or have a self‐limiting clinical course, infection is followed by a wide range of pathological responses, including hemophagocytic lymphohistiocytosis (HLH), chronic active EBV infection (CAEBV), and neoplasia, which can occur in both patients with immunodeficiency and individuals without clear immunodeficiency.^7^ The mortality rate of EBV‐associated HLH (EBV‐HLH) in children can be as high as 56%. In Asian countries, the prognosis of adults with EBV‐HLH is much worse: a previous study that included 61 patients of EBV‐HLH reported a 1‐year overall survival (OS) rate of only 25%.^8^, ^9^ CAEBV is the most common EBV infection in East Asia: in patients with EBV‐associated T/NK cell lymphoid tissue appreciation disease (LPD), which is associated with CAEBV, the survival rate is 44%. Currently, effective preventive measures against EBV infection do not exist, and available treatments for CAEBV and EBV‐HLH, mostly with rituximab, antiviral agents, and chemotherapeutic agents, remain unsatisfactory. Allogeneic hematopoietic stem cell transplantation is currently the only cure for the disease, but the treatment‐related mortality is high.^10^, ^11^

EBV may control antigen presentation directly or indirectly by downregulating the expression of HLA Classes I and II and reducing immune surveillance by virus‐specific CD4^+^ and CD8^+^ T cells.^12^ In EBV‐associated diseases, the underlying hypothesis is that different HLA Class I alleles differ in their ability to present viral peptides and that the host response to inhibit viral replication is modified by these differences, which ultimately affect EBV‐associated diseases.^13^ As EBV‐associated diseases are associated with different EBV‐infected lymphocyte types and because HLA Class I may affect the EBV‐infected lymphocyte types in peripheral blood mononuclear cells (PBMCs), this study aimed to explore the relationships between HLA alleles and EBV‐associated diseases.

## 2 METHODS

### 2.1 Sample cohort

The study cohort consisted of 269 consecutive patients with EBV‐related diseases (EBV‐seropositive group) and 213 EBV‐seronegative hematopoietic stem cell donors (EBV‐seronegative group) who visited the Beijing Friendship Hospital, Capital Medical University from January 2015 to December 2020, the hematopoietic stem cell donors as the control are not random. We retrospectively collected data on patient characteristics and parameters such as EBV DNA levels in PBMCs and plasma at the time of first admission. Furthermore, we retrospectively investigated EBV‐infected lymphocyte types in 107 patients with high numbers of EBV copies in circulating blood (EBV DNA level >5 × 10^2^ copies/ml in PBMCs).

### 2.2 DNA extraction and HLA genotyping

HLA genotyping involves PCR amplification of sample DNA using HLA gene‐specific PCR amplification primers, purification, and sequencing of the HLA gene amplification product using gene exon sequencing primers, and comparison with standard sequences to determine the HLA gene type. Genomic DNA was extracted from peripheral blood samples using Qiagen's Genomic DNA Extraction Kit according to the manufacturer's instructions. Two sets of samples were typed by polymerase chain reaction (PCR) using forward and reverse primers for HLA‐A, ‐B, ‐C, ‐DRB1, and ‐DQB1. The typed PCR products were directly sequenced using the method and kit (HLAssure SE A/B/C/DRB1/DQB1 locus SBT Kit) and HLA gene‐specific PCR amplification primers and HLA typing. The purified sequencing amplification products were sequenced using an ABI3730XL sequencer. The results obtained after sequencing were compared with the HLA standard types published in the IMGT database of international organizations (http://www.ebi.ac.uk/imgt/); the sequencing peak maps were analyzed and processed using specialized software such as Nomi 1.0, Chromas, and PyHLA to draw conclusions about the sample type based on the degree of match; the software uTYPE is used for HLA genotyping.

### 2.3 Identification of lymphocyte types infected by EBV

In Step (a), monocytes are extracted from human peripheral blood. In Step (b), said monocytes are sorted from human peripheral blood and magnetic beads capable of specifically screening CD3+ T lymphocytes, CD4+ T lymphocytes, CD8+ T lymphocytes, CD19+ B lymphocytes, or CD56+ NK cells are applied to obtain separate subpopulations of lymphocytes. In Step (c), purity is verified by flow cytometry and the criterion is quantitative reverse transcription‐polymerase chain reaction (qRT‐PCR) of the cells when the purity of the sorted cells is 90% or higher (including 90%) to reliably verify the presence of EBV infection. DNA is extracted from each of the different lymphocyte subpopulations described, and the EBV‐DNA copy number of each sorted cell is measured on 10 ng of DNA by applying the Zhongshan Daan Gene EBV Detection Kit and 7500 Fast Real‐Time Fluorescence PCR instrument to identify EBV‐infected cells and calculate the EBV copy number per 106 cells based on their copy number EBV copy number, which was defined as >500 per million cells.

### 2.4 Statistical analysis

Allelic frequencies (AFs) and haplotype frequencies (HFs) were calculated for HLA‐A, ‐B, ‐C, ‐DRB1, and ‐DQB1 loci using Arlequin 3.5.2.2 software. The EBV‐seropositive and EBV‐seronegative groups were compared using GraphPad software version 5.0. Pearson's *χ* ^2^ odds ratio (OR) reflects the strength of the association between exposure and disease, and the statistical significance threshold was set at *p* < .05. OR values were interpreted as follows: OR > 1, an increased risk of positive association between HLA genotype and EBV‐related diseases, with a high likelihood of the presence of alleles associated with susceptibility to an EBV‐related disease; OR < 1, a decreased risk of positive association between HLA genotype and EBV‐related diseases, with a low likelihood of presence of alleles associated with susceptibility to an EBV‐related disease or high likelihood of presence of a protective allele against an EBV‐related disease. Benjamini & Hochberg in 1995 proposed a method to determine the domain value of the *p* value by controlling the FDR (false discovery rate) to determine the domain value of *p* values. The method is as follows: Let there be a total of *m* candidate genes, and the *p* values of each gene are *p* (1), *p*(2),…,*p*(*m*), then if we want to control the FDR not to exceed *q*, we just need to find the largest positive integer *i* such that *p*(*i*) ≤ (i*q)/*m*. Then, pick the genes corresponding to *p*(1), *p*(2),…,*p*(*i*) as differentially expressed genes, so that the FDR is statistically guaranteed not to exceed *q* (e.g., *q* = 0.05 or 0.01).

## 3 RESULTS

A total of 482 peripheral blood samples, including 269 patients with EBV‐related disease (including 199 with EBV‐HLH, 32 with chronic active EBV infection [CAEBV], 38 with EBV‐associated LPD disease [EBV‐LPD], and 213 EBV seronegative haematopoietic stem cell donors) were included. The current study involved the detection of initial EBV DNA levels in PBMCs and plasma at first admission, together with the examination of HLA Class I and Class II molecular typing results to analyze the association between HLA and EBV‐associated diseases. The high‐resolution HLA typing showed that both groups had the highest AFs at A 11:01 (18.34%), A 24:02 (17.46%), and DQB1 03:01 (16.91%) (Table 1).

**Table 1:** Frequencies of HLA‐A, ‐B, ‐C, ‐DRB1, and ‐DQB1 alleles determined using high‐resolution analysis in the EBV‐seropositive and EBV‐seronegative groups

Allele frequency	EBV‐seropositive patients (*N*, %)		EBV‐seronegative donors (*N*, %)		*p*	Adjust *p*	OR	95% CI	
A*01:01	29	0.0541	21	0.0493	0.7718	0.9611	1.1031	0.6197	1.9635
A*02:01	59	0.1101	63	0.1479	0.097	0.5667	0.7127	0.4872	1.0426
A*02:06	28	0.0522	31	0.0728	0.2233	0.7592	0.7023	0.4144	1.1904
A*02:07	46	0.0858	32	0.0751	0.6346	0.9611	1.1559	0.7222	1.8499
A*11:01	96	0.1791	78	0.1831	0.9328	0.9611	0.9734	0.6997	1.3543
A*24:02	96	0.1791	72	0.169	0.7326	0.9611	1.0727	0.7665	1.5012
A*33:03	45	0.084	32	0.0751	0.6345	0.9611	1.1284	0.7037	1.8095
B*15:01	**43**	**0.0799**	**20**	**0.0469**	**0.0484**	**0.5667**	**1.7634**	**1.021**	**3.0457**
B*40:01	43	0.0799	30	0.0704	0.625	0.9611	1.1467	0.7063	1.8617
B*46:01	54	0.1004	38	0.0892	0.5826	0.9611	1.1392	0.7366	1.7617
B*51:01	41	0.0762	38	0.0892	0.48	0.9611	0.8423	0.5313	1.3355
C*01:02	74	0.1381	56	0.1315	0.7768	0.9611	1.0583	0.7287	1.5369
C*03:03	49	0.0914	40	0.0939	0.9112	0.9611	0.9709	0.6263	1.5053
C*03:04	53	0.0989	32	0.0751	0.21	0.7592	1.3511	0.8542	2.137
C*04:01	44	0.0821	23	0.054	0.098	0.5667	1.567	0.9305	2.6389
C*06:02	54	0.1007	44	0.1033	0.9149	0.9611	0.9727	0.639	1.4805
C*07:02	67	0.125	67	0.1573	0.1603	0.6813	0.7655	0.5311	1.1032
C*14:02	29	0.0541	30	0.0704	0.3439	0.9335	0.755	0.4457	1.279
DQB1*02:01	29	0.0539	21	0.0493	0.7722	0.9611	1.0988	0.6173	1.9558
DQB1*02:02	46	0.0855	38	0.0892	0.9085	0.9611	0.9546	0.6088	1.4969
DQB1*03:01	81	0.1506	82	0.1925	0.1	0.5667	0.7436	0.5306	1.042
DQB1*03:02	51	0.0948	28	0.0657	0.1239	0.6018	1.4886	0.9214	2.4048
DQB1*03:03	90	0.1673	68	0.1596	0.7929	0.9611	1.0576	0.7496	1.4922
DQB1*05:01	34	0.0632	24	0.0563	0.6847	0.9611	1.13	0.6593	1.9366
DQB1*05:02	46	0.0855	29	0.0681	0.335	0.9335	1.2799	0.7895	2.0751
DQB1*05:03	31	0.0576	19	0.0446	0.3844	0.9335	1.3098	0.7291	2.3528
DQB1*06:01	44	0.0818	33	0.0775	0.9049	0.9611	1.0607	0.6627	1.6978
DQB1*06:02	**30**	**0.0558**	**40**	**0.0939**	**0.0249**	**0.5667**	**0.5699**	**0.3486**	**0.9317**
DRB1*03:01	30	0.0558	21	0.0493	0.7723	0.9611	1.1389	0.6423	2.0194
DRB1*07:01	57	0.1059	45	0.1056	1	1.0000	1.0033	0.6636	1.5169
DRB1*08:03	41	0.0762	26	0.061	0.375	0.9335	1.2692	0.7631	2.1108
DRB1*09:01	81	0.1506	62	0.1455	0.8556	0.9611	1.0406	0.7273	1.4889
DRB1*12:02	29	0.0539	28	0.0657	0.4924	0.9611	0.8099	0.474	1.3836
DRB1*15:01	42	0.0781	48	0.1127	0.0746	0.5667	0.6668	0.4315	1.0305

Abbreviations: CI, confidence interval; EBV, Epstein‐Barr virus; OR, odds ratio.

We found that the frequency of DRB1*11, among the HLA‐A, ‐B, ‐C, ‐DRB1, and ‐DQB1 alleles, differed significantly between the EBV‐seropositive and EBV‐seronegative groups: the frequency of DRB1*11 was 4.09% in the EBV‐seropositive group and 7.04% in the EBV‐seronegative group (OR = 0.56, 95% CI = 0.32–0.99); OR < 1 suggests a reduced risk of EBV‐associated diseases in the HLA‐DRB1*11 population (Table 2). The high‐resolution frequency analysis of the HLA‐A, ‐B, ‐C, ‐DRB1, and ‐DQB1 alleles in EBV‐seropositive and EBV‐seronegative groups showed statistically significant differences in HLA‐B*15:01 and HLA‐DQB1*06:02 between the groups: B*15:01, 7.99% versus 4.69% (OR = 1.76, 95% CI = 1.02–3.05; OR > 1, DQB1*06:02, 5.58% versus 9.39% (OR = 0.57, 95%; CI = 0.35–0.93 (Table 1). The frequency of DRB1*11 was 4.09% in the EBV‐seropositive group and 7.04% in the EBV‐seronegative group (OR = 0.56, 95% CI = 0.32–0.99) (Table 2). OR < 1 suggests a reduced risk of EBV‐associated diseases in the HLA‐DQB1*06:02 population) but did not reach statistical significance after correction of the *p* value (Table 1).

**Table 2:** Frequencies of HLA‐A, ‐B, ‐C, ‐DRB1, and DQB1 alleles in the EBV‐seropositive and EBV‐seronegative groups

	EBV‐seropositive patients		EBV‐seronegative donors						
Allele frequency	*N*	%	*N*	%	*p*	Adjust *p*	OR	OR	95% CI
A*01	29	5.41%	21	4.93%	0.7718	1.0000	1.1031	0.6197	1.9635
A*02	153	28.54%	140	32.86%	0.1586	0.7092	0.8161	0.6194	1.0751
A*11	104	19.40%	83	19.48%	1	1.0000	0.9949	0.7214	1.372
A*24	105	19.59%	76	17.84%	0.5074	1.0000	1.1219	0.8089	1.5561
A*33	46	8.58%	32	7.51%	0.6346	1.0000	1.1559	0.7222	1.8499
B*13	51	9.48%	38	8.92%	0.8231	1.0000	1.0693	0.6882	1.6614
B*15	82	15.24%	59	13.85%	0.5824	1.0000	1.1186	0.779	1.6061
B*35	31	5.76%	23	5.40%	0.8882	1.0000	1.0713	0.615	1.8663
B*40	74	13.75%	57	13.38%	0.9247	1.0000	1.0324	0.7121	1.4969
B*46	54	10.04%	38	8.92%	0.5826	1.0000	1.1392	0.7366	1.7617
B*51	51	9.48%	41	9.62%	1	1.0000	0.9834	0.6382	1.5152
C*01	79	14.74%	58	13.62%	0.6433	1.0000	1.0968	0.7609	1.581
C*03	132	24.63%	91	21.36%	0.249	0.7092	1.2028	0.8877	1.6298
C*04	50	9.33%	30	7.04%	0.2398	0.7092	1.358	0.8473	2.1765
C*06	54	10.07%	44	10.33%	0.9149	1.0000	0.9727	0.639	1.4805
C*07	81	15.11%	78	18.31%	0.1909	0.7092	0.7943	0.5649	1.1168
C*08	36	6.72%	33	7.75%	0.6152	1.0000	0.8575	0.525	1.4003
C*14	32	5.97%	34	7.98%	0.2483	0.7092	0.732	0.4438	1.2075
C*15	31	5.78%	24	5.63%	1	1.0000	1.0282	0.594	1.78
DQB1*02	75	13.94%	59	13.85%	1	1.0000	1.0076	0.6977	1.4552
DQB1*03	226	42.01%	180	42.25%	0.9477	1.0000	0.99	0.7653	1.2806
DQB1*04	30	5.58%	27	6.34%	0.6805	1.0000	0.8727	0.5105	1.4919
DQB1*05	112	20.82%	72	16.90%	0.1375	0.7092	1.2926	0.9313	1.7943
DQB1*06	95	17.66%	88	20.66%	0.2478	0.7092	0.8237	0.5965	1.1374
DRB1*03	30	5.58%	21	4.93%	0.7723	1.0000	1.1389	0.6423	2.0194
DRB1*04	80	14.87%	52	12.21%	0.2579	0.7092	1.2563	0.8635	1.8277
DRB1*07	57	10.59%	45	10.56%	1	1.0000	1.0033	0.6636	1.5169
DRB1*08	46	8.55%	32	7.51%	0.6346	1.0000	1.1512	0.7193	1.8423
DRB1*09	81	15.06%	62	14.55%	0.8556	1.0000	1.0406	0.7273	1.4889
DRB1*11	**22**	**4.09%**	**30**	**7.04%**	**0.0457**	**0.7092**	**0.5628**	**0.3197**	**0.9907**
DRB1*12	37	6.88%	42	9.86%	0.0989	0.7092	0.6752	0.4256	1.0712
DRB1*14	56	10.41%	33	7.75%	0.179	0.7092	1.3836	0.882	2.1706
DRB1*15	53	9.85%	56	13.15%	0.1244	0.7092	0.722	0.4844	1.0763

Abbreviations: CI, confidence interval; EBV, Epstein‐Barr virus; OR, odds ratio.

Considering that polymorphisms in HLA Class I molecules may be associated with viral recognition by immune cells and that EBV‐infected lymphocyte subtypes influence the prognosis of an EBV‐associated disease, it is currently believed that patients with EBV‐infected T cells/NK cells have a poorer prognosis.^14^, ^15^ We further analyzed the results of 27 patients with an EBV‐associated disease showing the HLA‐B*15:01 genotype and 80 patients with an EBV‐associated disease of other genotypic EBV‐infected lymphocyte subgroups. The patients with an EBV‐associated disease in other genotypic subgroups had the following phenotypes: detectable EBV‐infected B‐cell phenotype, 22 patients (27.5%); detectable EBV‐infected T‐cell phenotype, 6 patients (7.5%); detectable EBV‐infected NK‐cell phenotype, 22 patients (27.5%); detectable EBV‐infected NK/B cell phenotype, 1 patient (1.25%); and detectable EBV‐infected NK/T cell phenotype, 6 patients (7.5%); EBV‐infected multilineage phenotype, 23 patients (28.7%). Among patients with EBV‐related diseases of the HLA‐B*15:01 genotype, the following phenotypes were detected: EBV‐infected T‐cell phenotype, 1 patient (3.7%); EBV‐infected NK‐cell phenotype, 3 patients (11.11%); EBV‐infected NK/B‐cell phenotype, 4 patients (14.81%); EBV‐infected NK/T‐cell phenotype, 5 patients (18.52%); and EBV‐infected multilineage cells, 13 patients (48.15%). We found that EBV‐infected patients with HLA‐B*15:01 predominantly showed the EBV‐infected multilineage NK/T‐cell phenotype, and those with an EBV‐associated disease without HLA‐B*15 showed the EBV‐infected B‐cell phenotype; however, the other genotypes predominantly showed EBV‐infected multilineage B‐cell and NK‐cell phenotypes. EBV‐infected lymphocyte subpopulations in patients with EBV‐associated diseases were significantly different between the HLA‐B*15:01 and other genotype groups (*p* < .05). Therefore, it is considered that patients with EBV‐related diseases with HLA‐B*15:01 predominantly show infected NK and T cells and rarely show infected B cells (Table 3).

**Table 3:** Epstein‐Barr virus (EBV)‐infected cell types according to genotype subgroups

EBV‐infected lymphocyte cell types		Group		
No. (%)		Other genotype subgroups	HLA‐B*15:01 group	Total
B cell	*n*	22	0	22
	%	27.50	0.00	20.56
T cell	*n*	6	1	7
	%	7.50	3.70	6.54
NK cells	*n*	22	3	25
	%	27.50	11.11	23.36
T/B cells	*n*	0	1	1
	%	0.00	3.70	0.93
NK/B cells	*n*	1	4	5
	%	1.25	14.81	4.67
NK/T cells	*n*	6	5	11
	%	7.50	18.52	10.28
Multiple	*n*	23	13	36
	%	28.75	48.15	33.64
Total	*n*	80	27	107

## 4 DISCUSSION

EBV was first identified in 1964 by Epstein and Barr in African children with Burkitt lymphoma (BL), and it is the only lymphoid follicular virus in the gamma subfamily of the herpesvirus family that causes infection in humans. After primary infection, the viral genes are present in certain tissues or cells and do not lead to the development of infectious viral and clinical symptoms.^16^ However, some patients can still develop highly lethal diseases including EBV‐LPD and EBV‐HLH, and currently available treatments for these diseases are unsatisfactory. The pathogenesis of EBV‐related diseases is still being explored due to differences in morbidity and mortality between Asia and other regions. HLA Classes I and II are highly polymorphic molecules that present antigens of pathogens to the immune system for recognition by T cells. HLA Class II plays a key role in EBV infection because it is required for the initial stages of viral infection of B cells, which are the sites of viral latency. Specific HLA Class II alleles are associated with increased or decreased susceptibility to EBV infection, which is also associated with their ability to bind to EBV gp42.^9^ In contrast, although HLA Class I is not required for viral entry, polymorphisms in HLA Class I alleles are associated with the development and severity of infectious mononucleosis.^10^ Specific HLA class I alleles have been associated with several EBV‐related malignancies, including nasopharyngeal carcinoma, Hodgkin's lymphoma, and post‐transplant lymphoproliferative disorders.^11^ Thus, although EBV has evolved to require HLA Class II molecules to infect humans, it is thought that both Class I and Class II molecules affect the susceptibility of infected humans to EBV‐associated diseases.^8^ We, therefore, explored protection‐ and susceptibility‐related subtypes in HLA alleles by comparing the HLA types in Chinese patients with EBV‐associated diseases versus healthy donors. To the best of our knowledge, this study includes the highest number of analyses on HLA Classes I and II molecules in Chinese patients with EBV infection so far.

In this study, we demonstrated that the HLA‐B*15:01 genotype group was associated with a higher risk of an EBV‐related disease than was the EBV‐seronegative group and found a decreased likelihood of presence of HLA‐DRB1*11 and HLA‐DQB1*06:02 in the former group, but did not reach statistical significance after correction of the *p* value. According to domestic reports on HLA genotypes, the current frequency of B*15:01 in the population is approximately 4.69%. In this study, the frequency of HLA‐B*15:01 genotype was found to be higher in the EBV‐seropositive group than in the population in general, and the frequency of DQB1*06:02 in the EBV‐seronegative group in this study was also significantly lower than the domestically reported frequency (8.68%); our findings on B*15:01 and DQB1*06:02 frequencies have not been reported before.^12^ The B*15:01 genotype, which was observed in humans in the current study, may also be associated with other viral infection states, with the frequency of B*15:01 being significantly higher in the hepatitis C virus (HCV)‐infected group than in the non‐HCV‐infected group (OR = 1.561, *p* = .010).^13^, ^14^ Investigators have speculated that it may affect the ability of virus to bind to HLA antigens, and it has also been suggested that it may be associated with hepatitis B virus clearance and chronicity. Our findings suggest that this genotype can increase the risk of EBV‐related diseases.^15^ However, DQB1*06:02 has been reported less frequently and is thought to be associated with a genetic sleep disorder.^16^

Previous reports on HLA genotypes for EBV infection status in Asia are scarce. HLA Class I molecular polymorphisms may be associated with genetic variation in T cell responses, which may influence the course of EBV infection and serum levels of virus, and with the development and severity of infectious mononucleosis.^17^ Previous reports suggest that healthy donors who carried HLA‐A1 genotypes showed a reduced incidence of seropositivity and that HLA‐A10, ‐A29, and ‐B15 were less prevalent in EBV‐seronegative than EBV‐seropositive populations.^18^ According to a previous study, HLA‐C*04:01 was associated with an increased risk of infectious mononucleosis, whereas HLA‐C*02:02 was associated with a reduced risk in students with asymptomatic seroconversion to EBV.^19^ However, the above cohort results were obtained from the European population, and our results suggest that in the Chinese population, the HLA‐B*15:01 genotype is associated with an increased disease risk, which is in turn associated with a high EBV‐related mortality rate.

HLA Class II molecules present extracellular antigens to T cells, and because EBVgp 42 enters B cells by interacting with HLA Class II molecules, B cells with different HLA Class II molecular sequences may show differential susceptibility to EBV infection. In a previous in vitro study on the transient expression of HLA‐DQ in a B cell line that lacks HLA Class II expression, cells expressing HLA‐DQ3.3 (α*0301 × β*03032) were found to be less susceptible to EBV infection, whereas those expressing HLA‐DQ2 (a*0501 × β*0201) were more susceptible to viral infection.^20^ Our results suggest a reduced risk of EBV‐associated lethal diseases in the Chinese population that shows the expression of HLA‐DQB1*06:02.

B‐cell type infections were observed in immunocompromised patients as well as in patients with ASHEBV, IM, EBV + B‐LPDs, and EBV + BCLs. Prevailing clinical treatment options include the watch/wait approach and steroidal and/or antiviral therapy.^17^ In contrast, T‐cell, NK‐cell, and multicell EBV infections in immunocompetent hosts are highly suggestive of EBV‐positive T/NK‐cell diseases and require immediate diagnosis and prompt treatment. The literature suggests that the 5‐year OS for CAEBV was as follows: T‐cell type, 59%; NK‐cell type, 87%.^21^ An important role of HLA antigen expression has been reported to be a coreceptor for EBV entry into B cells. HLA antigen on T or NK cells may play a role in the pathogenesis of EBV‐HLH and CAEBV, as its expression is known to be upregulated in vivo and in vitro by several stimulants and because EBV‐infected T or NK cells in patients with CAEBV and EBV‐HLH express the HLA antigen. It has, therefore, been hypothesized that HLA alleles may affect EBV‐infected cell subsets; however, studies verifying this hypothesis are lacking. We further analyzed the results of 27 patients with EBV‐associated diseases of the HLA‐B*15:01 genotype and 80 patients with EBV‐associated diseases of other genotypic EBV‐infected lymphocyte subgroups. This study suggests that the B*1501 group is much more represented in patients with T‐cell, NK‐cell, and multi‐cell EBV infections than in those with other genotypic subgroups, suggesting that patients with the B*1501 genotype are more likely to have non‐B‐cell infections and that patients with the B*15:01 genotype should be examined for the predominant lymphocyte type showing EBV infection and for the early initiation of intervention. Our findings suggest a risk of HLA‐associated susceptibility genes in patients with EBV infection. Further studies with larger sample sizes are needed to confirm these results and to determine the role of HLA alleles and haplotypes in the association with EBV infection.

## AUTHOR CONTRIBUTIONS

Yini Wang and Zhao Wang designed the experiments. Dina Suolitiken performed the experiments, analyzed the data, and wrote the paper with assistance from all the other authors. Zhili Jin coordinated the clinical materials. All authors participated in the revision and approval of the manuscript.

## CONFLICT OF INTEREST

The authors declare no conflict of interest.

## ETHICS STATEMENT

All protocols that involved human participants in this study complied with the institutional ethical requirements (Number of approval letter 2020‐P2‐096‐01).

## Data Availability

The data of our patients can be obtained from the medical records in the Department of Medical Records at Beijing Friendship Hospital. These data can be released with the consent of the patients and are available from the corresponding author upon reasonable request.
